# To center or not to center? Investigating inertia with a multilevel autoregressive model

**DOI:** 10.3389/fpsyg.2014.01492

**Published:** 2015-01-06

**Authors:** Ellen L. Hamaker, Raoul P. P. P. Grasman

**Affiliations:** ^1^Methodology and Statistics, Faculty of Social and Behavioural Sciences, Utrecht UniversityUtrecht, Netherlands; ^2^Psychological Methods, University of AmsterdamAmsterdam, Netherlands

**Keywords:** centering, autoregressive models, multilevel models, dynamics, inertia

## Abstract

Whether level 1 predictors should be centered per cluster has received considerable attention in the multilevel literature. While most agree that there is no one preferred approach, it has also been argued that cluster mean centering is desirable when the within-cluster slope and the between-cluster slope are expected to deviate, and the main interest is in the within-cluster slope. However, we show in a series of simulations that if one has a multilevel autoregressive model in which the level 1 predictor is the lagged outcome variable (i.e., the outcome variable at the previous occasion), cluster mean centering will in general lead to a downward bias in the parameter estimate of the within-cluster slope (i.e., the autoregressive relationship). This is particularly relevant if the main question is whether there is on average an autoregressive effect. Nonetheless, we show that if the main interest is in estimating the effect of a level 2 predictor on the autoregressive parameter (i.e., a cross-level interaction), cluster mean centering should be preferred over other forms of centering. Hence, researchers should be clear on what is considered the main goal of their study, and base their choice of centering method on this when using a multilevel autoregressive model.

Longitudinal data are characterized by a nested structure, in which occasions are clustered within individuals. While such data are traditionally analyzed using repeated measures ANOVA, this approach is restrictive in that it requires an equal number of observations for each participant. A further limitation associated with repeated measures ANOVA is that the results pertain to the aggregate and may not be meaningful for any particular individual. A more sophisticated approach—which overcomes these limitations—is *multilevel modeling* (Singer and Willett, 2003; Hox, 2010; Snijders and Bosker, 2012; also known as mixed modeling, see Verbeke and Molenberghs, 2000; hierarchical modeling, see Raudenbush and Bryk, 2002; or random-effects modeling, see Laird and Ware, 1982): This approach can be used for (highly) unbalanced longitudinal data, and it allows for individual trajectories over time. The latter implies we can study between-person (or interindividual) differences in within-person (or intraindividual) patterns of change.

It is not uncommon for the residuals of a longitudinal multilevel model to be autocorrelated, meaning that residuals are related to each other over time. Failing to account for this may bias the estimates of the standard errors, and as a result affect the inferences based on them. Therefore, multilevel software packages include the option to control for autocorrelation through specifying diverse structures for the errors, such as a Toeplitz matrix, or a first order autoregressive process. Alternatively, autocorrelation can be modeled explicitly through the inclusion of the lagged outcome variable (that is, the outcome variable at the previous occasion) as a covariate. Such models have been referred to as (prospective) change models (e.g., Larson and Almeida, 1999), and are used to investigate the—potentially causal—effect of a (lagged) predictor on the outcome variable, while “controlling” or “adjusting” for the previous level of the outcome variable (e.g., Bolger and Zuckerman, 1995; Gunthert et al., 2007; Moberly and Watkins, 2008; Henquet et al., 2010).

While autocorrelation is typically considered a nuisance in longitudinal multilevel modeling, there are a few multilevel studies that focus specifically on the autoregressive relationship between consecutive observations, and on individual differences therein (cf., Suls et al., 1998; Rovine and Walls, 2006; Kuppens et al., 2010; Koval and Kuppens, 2012; Wang et al., 2012; Brose et al., 2014). The interest in an individual's autoregressive parameter comes from the fact that this parameter is related to the time it takes the individual to recover from a perturbation and restore equilibrium: While an autoregressive parameter close to zero implies that there is little carryover from one measurement occasion to the next and recovery is thus instant, an autoregressive parameter close to one implies that there is considerable carryover between consecutive measurement occasions, such that perturbations continue to have an effect on subsequent occasions. For this reason, the autoregressive parameter can also be considered as a measure of *inertia* or *regulatory weakness*.

Empirical studies have shown that individual differences in inertia in emotions and affect are positively related to neuroticism and depression, in that people higher on neuroticism or depression take longer to restore equilibrium than others (Suls et al., 1998; Kuppens et al., 2010; Wang et al., 2012). In addition, women tend to have higher inertia than men in both their daily affect (Wang et al., 2012), and their daily drinking behavior (Rovine and Walls, 2006). In a prospective study, Kuppens et al. (2012) showed that affective inertia at age 9–12 was predictive of the onset of depression two and a half years latter, corresponding to the idea that high inertia is reflective of a maladaptive regulation mechanism. Similarly, Wang et al. (2012) showed that inertia is positively related to later detrimental health outcomes. Furthermore, inertia has been shown to be related—but not identical—to rumination (Koval et al., 2012) and perseverative thoughts (Brose et al., 2014), and is positively related to depression even after these related characteristics are taken into account. Taken together, these studies show that inertia is a meaningful individual characteristic that is reflective of a maladaptive regulatory mechanism that is associated with both current *and* future well-being.

To model individual differences in inertia, the above studies all relied on multilevel modeling based on a first-order autoregressive process: In this model, the level 1 predictor is formed by the lagged outcome variable, and its random slope thus represents individual differences in inertia. A pressing question in this context is whether the autoregressive predictor should be centered per person or not. This is a rather fundamental issue, as it is well-known from the multilevel literature that the centering method used for a level 1 predictor (i.e., no centering, centering with the grand mean, or centering per cluster), affects the results (cf. Kreft et al., 1995; Raudenbush and Bryk, 2002; Hox, 2010; Snijders and Bosker, 2012). The consensus seems to be that there is no one preferred method and that the choice should depend on the specific situation and the research question (cf. Kreft et al., 1995; Nezlek, 2001; Snijders and Bosker, 2012). One such specific situation is described by Raudenbush and Bryk (2002), who indicate that if the within-cluster and between-cluster slopes differ, centering per cluster should be preferred, because failing to do so will lead to results that are “uninterpretable” (p. 135). Furthermore, Enders and Tofighi (2007) argue that if there is a clear interest in the within-cluster slope, centering per cluster is recommendable.

With this latter advice in mind, centering the lagged autoregressive predictor per person seems the right approach, because: (a) we are interested in the within-person slope; and (b) we expect the within-person slope to differ from the between-person slope.^1^ The aim of the current paper is therefore to investigate whether the advice formulated by Raudenbush and Bryk (2002) and Enders and Tofighi (2007) also applies to the multilevel autoregressive model with a random slope that represents individual differences in inertia. To this end, we begin by presenting the multilevel autoregressive model and discuss its interpretation. To make the model compatible with standard multilevel software, we discuss two parameterizations—based on different centering methods—and we show through an empirical application that these lead to different results for the inertia parameter. In the second section we draw from several key publications in the multilevel literature and discuss the effects of centering a level 1 predictor. The third section contains simulations based on the standard multilevel model to verify some of the claims made in the literature. Additionally, we simulate the multilevel autoregressive model to investigate how centering affects the estimation of inertia. In the fourth section we apply the insights obtained from the simulation study to the empirical data set. We end by presenting recommendations to the researcher interested in studying inertia using the multilevel autoregressive model, either with or without level 2 predictors.

## 1. Multilevel autoregressive model

Many applications of longitudinal multilevel modeling consist of modeling deterministic trajectories over time, for instance a linear or quadratic trend. While such models are extremely useful for studying developmental processes (cf., Curran and Bauer, 2011), they may be less useful when the longitudinal data comprise daily affective or symptom measurements, or affective ratings in an observation study: Then the interest may be not so much in overall trends (as they are likely to be absent from the data), but rather in the dynamics of a stationary process, that is, a process that is characterized by changes *over time*, while these changes are not directly a *function of time*. A promising model for this purpose is the multilevel autoregressive model, which has been successfully applied in an increasing number of studies (e.g., Suls et al., 1998; Rovine and Walls, 2006; Kuppens et al., 2010; Koval and Kuppens, 2012; Wang et al., 2012; Brose et al., 2014).

We begin this section by presenting the multilevel autoregressive model using a parametrization that we consider to be most useful from a substantive viewpoint. However, since this parametrization is not compatible with standard multilevel software, we also present two alternative parametrization of this model, and discuss their advantages and disadvantages. We apply both parameterizations to an empirical data set consisting of daily measurement of positive and negative affect.

### 1.1. A model to study individual differences in mean and inertia

Let *y*_*ti*_ be the observation for individual *i* at occasion *t*, for instance the person's negative affect or self-esteem measured at a daily basis, with *i* = 1, …, *N* and *t* = 1, …, *T*_*i*_. The most basic model for such nested data would be a model which allows for individual differences in means. At level 1 the observations are then modeled as

(1) $$y_{ti}=\mu_{i}+a_{ti}$$

where μ_*i*_ represents the individual's mean score, which can be interpreted as his/her trait score or equilibrium, while *a*_*ti*_ is the individual's temporal deviation from this equilibrium; and at level 2 the individual means are then modeled as

(2) $$\mu_{i}=\mu +u_{0i}$$

where μ is the grand mean, and *u*_0*i*_ is the individual's deviation from the grand mean. These deviations are assumed to be normally distributed, that is, *u*_0*i*_ ~ *N*(0, σ^2^_*u*0_).^2^

If repeated measures are taken (relatively) close in time, the current measurement is likely to be predictable from the preceding measurement. That is, the individual's deviation from his/her equilibrium at a particular occasion is likely to affect the deviation at the next occasion, which can be expressed as a first order autoregressive model, that is,

(3) $$a_{ti}=\phi_{i}a_{t\;-\;1,i}+e_{ti}$$

where the residuals *e*_*ti*_ are independently and identically distributed, with *e*_*ti*_ ~ *N*(0, σ^2^_*e*_). This residual *e*_*ti*_ can be thought of as representing everything that influences the process under investigation: For instance, if we are measuring negative affect, factors that are likely to influence this process include the occurrence of negative or stressful events, the appraisal of these events and the associations and memories that they trigger, but also psychophysiological factors like caffeine or alcohol consumption, et cetera.

The autoregressive parameter ϕ_*i*_ relates the outcome variable to itself at the preceding occasion, and thus represents the *inertia* of the person. For an autoregressive process to be stationary, the autoregressive parameter has to lie between −1 and 1 (e.g., Hamilton, 1994). Note however that this does not imply that the autoregressive parameter is truly restricted to this range: Values larger than 1 (or smaller than −1) are possible, but the resulting process would no longer be a stationary process. In psychological research, this parameter typically lies somewhere between 0 and 0.6 (e.g., Rovine and Walls, 2006; Wang et al., 2012), and we are therefore not concerned about boundary constraints when estimating this model.

The individual differences in the autoregressive parameter can be modeled at level 2 as

(4) $$\phi_{i}=\phi +u_{1i}$$

where ϕ denotes the average autoregressive parameter across people, and *u*_1*i*_ denotes the individual's deviation from this average, with *u*_1*i*_ ~ *N*(0, σ^2^_*u*1_). Furthermore, the individuals' means and their autoregressive parameters may be correlated, as represented by the covariance between *u*_0*i*_ and *u*_1*i*_, which is denoted as σ_*u*0, *u*1_. Wang et al. (2012) for instance found a significant positive correlation of 0.40 between the individuals' means μ_*i*_ and their autoregressive parameters ϕ_*i*_ based on daily measurements of negative affect.

### 1.2. Making the model compatible with standard multilevel software

The model in Equations 1–4 represents the multilevel autoregressive model, where Equations 1 and 3 form level 1, while Equations 2 and 4 form level 2. However, most multilevel software packages do not allow for formulating a level 1 model using more than one equation. We consider two solutions for this.

The first solution consists of specifying the model at level 1 as

(5) $$y_{ti}=c_{i}+\phi_{i}^{n}y_{t\;-\;1,i}+e_{ti},$$

and at level 2 as

(6) $$c_{i}\;=\gamma_{00}^{n}+u_{0i}^{n}$$

(7) $$\phi_{i}^{n}\;=\gamma_{10}^{n}+u_{1i}^{n},$$

where the superscript *n* indicates that in this approach *no centering* (NC) was used (i.e., the raw data were used). The relationship between the model specified in Equations 1–4 and the model specified in Equations 5–7 is shown in Appendix 1; however, while the multilevel models presented here are *structurally* the same, the current formulation is based on the assumption that *c*_*i*_ is normally distributed, which necessarily implies that μ_*i*_ will not have a normal distribution (as it is a function of *c*_*i*_ and ϕ_*i*_, see Appendix 1). This is detrimental, as we are typically interested in μ_*i*_ as representing an individual's average or trait score, and assume these trait scores to be normally distributed in the population. In contrast, *c*_*i*_ is a rather arbitrary score (i.e., the expected score when the individual scored zero on the preceding occasion), that is of limited (or no) substantive interest, and for which we do not have a particular distributional expectation. Also, if we are interested in including predictors at level 2, we would prefer to use these as predictors of μ_*i*_, rather than of *c*_*i*_.

Therefore, we consider a second solution, which is based on using the individually centered lagged autoregressive predictor (*y*_*t* − 1, *i*_ − μ_*i*_), such that the model at level 1 is

(8) $$y_{ti}=\mu_{i}+\phi_{i}^{c}(y_{t\;-\;1,i}-\mu_{i})+e_{ti}$$

and at level 2 it is

(9) $$\mu_{i}=\gamma_{00}^{c}+u_{0i}^{c}$$

(10) $$\phi_{i}^{c}\;=\gamma_{10}^{c}+u_{1i}^{c}$$

where the superscript *c* implies that the level 1 predictor was subjected to *cluster mean centering* (CMC; also referred to as within-group or within-person centering). The advantage of the current approach over the previous one is that it results in μ_*i*_ and ϕ_*i*_ being the random coefficients that are subsequently modeled at level 2. However, it also presents us with a catch-22: To center the lagged predictor, we need an estimate of μ_*i*_, which we actually need to estimate using this model. We will consider several solutions to this problem in our simulations, including the use of the sample mean per person.

### 1.3. Application: part 1

To investigate whether the two approaches proposed above lead to the same or different results for the inertia parameter, we apply the two parameterizations of the multilevel autoregressive model to an empirical data set that was obtained as part of the Dynamics of Dyadic Interactions Project at the University of California, Davis (Ferrer and Widaman, 2008; Ferrer et al., 2012). The data used here consist of daily measurements of relationship specific positive and negative affect. We analyzed these data for men and women separately (sample sizes 193 and 192, respectively), using multilevel autoregressive models with random intercepts (i.e., *c*_*i*_, based on NC) or means (i.e., μ_*i*_, based on CMC), and random autoregressive parameters (ϕ_*i*_). The estimates for the fixed effects parameters γ_00_ and γ_10_ are presented in Table 1.

**Table 1:** **Results for multilevel autoregressive model (with random effects)**.

				**NC**		**CMC**
Males	PA	γ_00_	2.167	[2.044, 2.290]	3.518	[3.425, 3.611]
		γ_10_	0.387	[0.357, 0.417]	0.353	[0.322, 0.384]
	NA	γ_00_	0.971	[0.923, 1.020]	1.344	[1.300, 1.389]
		γ_10_	0.268	[0.235, 0.301]	0.242	[0.208, 0.275]
Females	PA	γ_00_	2.220	[2.095, 2.346]	3.491	[3.392, 3.590]
		γ_10_	0.370	[0.340, 0.399]	0.341	[0.311, 0.370]
	NA	γ_00_	0.978	[0.935, 1.021]	1.348	[1.304, 1.392]
		γ_10_	0.255	[0.222, 0.288]	0.225	[0.192, 0.258]

Estimates for the fixed effects parameters in a multilevel autoregressive model (with random intercept and slope). The 95% confidence intervals are given between brackets. Estimation was based on using NC or CMC (with the sample means) for the lagged autoregressive predictor. Fixed effects are: (a) γ_00_, which represents the averaged intercept when using NC, or the grand mean when using CMC; and (b) γ_10_, which represents the averaged (i.e., fixed effects) autoregressive parameter.

It shows that the parameter estimates obtained with the two models are not identical. This is not surprising as we are already aware that the two parameterizations differ with respect to the meaning of γ_00_. However, it also shows that the parameter estimates for γ_10_—which represents the average inertia in both parameterizations—differ from each other. Especially when considering relationship specific PA in males, it can be seen that CMC and NC lead to estimates of the inertia that are not covered by the 95% confidence interval of the alternative parametrization (implying these estimates are relatively different).

The question thus arises, which approach should be preferred—NC or CMC—when the interest is in obtaining an appropriate estimate of the average autoregressive parameter. As this touches upon the more general topic of whether level 1 predictors should be centered or not in multilevel models, we first consult the multilevel literature with respect to centering level 1 predictors.

## 2. To center or not to center: a persisting question in multilevel modeling

**Figure 1:**
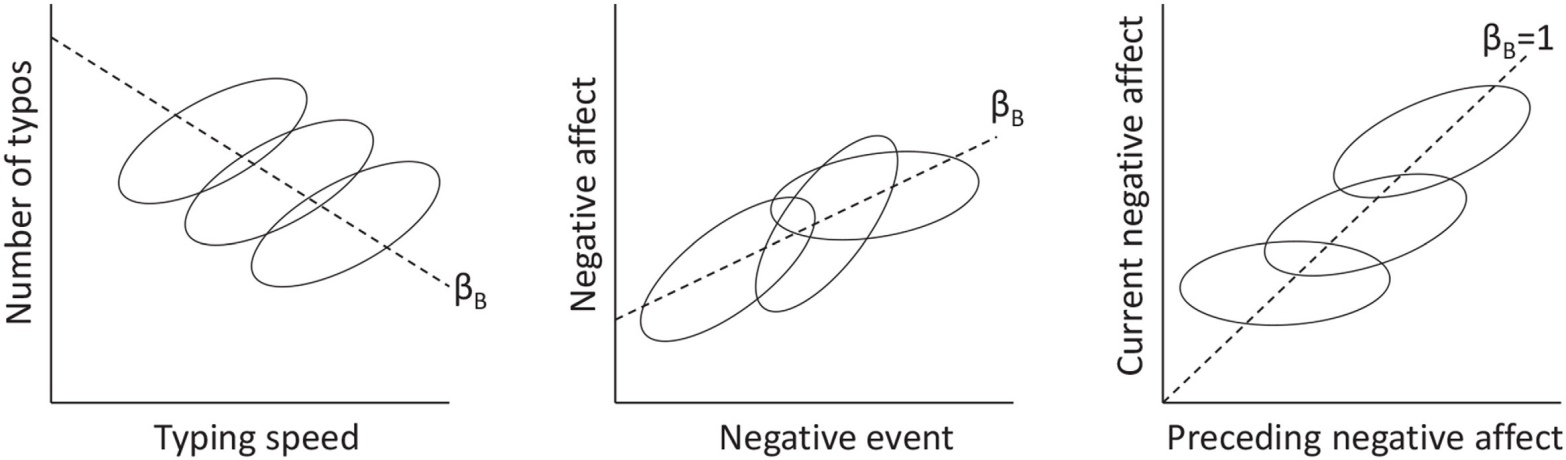
**Illustration of within-person and between-person relationships between two variables**. Each ellipse represents the data from a single person. Dashed lines represent the between-person slope (i.e., β_*B*_), which may have a different sign as the within-person slope (**Left panel**), may be similar to the (average or fixed) within-person slope (**Middle panel**), or may be larger than the (average or fixed) within-person slope (**Right panel**).

Centering a level 1 predictor in multilevel modeling is a complicated affaire. While there are several sources that provide excellent coverage of this topic (e.g., Kreft et al., 1995; Snijders and Bosker, 2012), it still seems to create much confusion, especially amongst the more novice users. A fundamental issue when dealing with a level 1 predictor is the fact that the relationship between a predictor and an outcome variable may differ across levels. For instance, consider the hypothetical example in the left panel of Figure 1, representing the relationship between typing speed and number of typos. This relationship is likely to be positive within individuals (i.e., at level 1), in that a person tends to make more mistakes if he/she types faster. However, the relationship across individuals (i.e., at level 2) is likely to be negative, because individuals who tend to type fast *on average*, also tend to be more experienced and therefore make fewer mistakes *on average* (cf. Hamaker, 2012; see also Nezlek, 2001; Enders and Tofighi, 2007; Kievit et al., 2013).

In this section we discuss the effects of different centering methods, when there are different slopes at the two levels. Our main interest is in obtaining an appropriate estimate for the within-cluster slope, as this is most informative with respect to the within-person process. To facilitate the transition to the multilevel autoregressive model, we will present the issue based on repeated measures within individuals (rather than individuals organized in groups). In following Raudenbush and Bryk (2002) and Enders and Tofighi (2007), we begin by considering models with a fixed slope only. Subsequently, we discuss contextual models, in which the cluster means are included as a predictor at level 2. Then we discuss extensions that allow for random slopes. We end this section by speculating on the effects of centering in the context of the multilevel autoregressive model.

### 2.1. The within-cluster and between-cluster slopes in multilevel data with a fixed slope

Suppose that *x*_*ti*_ is the predictor, such as typing speed or the occurrence of a negative event, and that *y*_*ti*_ is the outcome variable, such as number of typos or negative affect. Let *i* = 1, …, *N* denote the individual, and *t* = 1, …, *T*_*i*_ denote the measurement occasion within individual *i*. Raudenbush and Bryk (2002) discuss how to obtain estimates of the between-person slope, relating the trait scores on the outcome variable to the trait scores on the predictor, and of the averaged or pooled within-person slope, describing the process that operates within individuals, using ordinary least squares (OLS). To this end, we first need the individual means on the predictor and the outcome variable, that is,

(11) $${\bar{x}}_{\cdot i}=\frac{1}{T_{i}}\sum_{i\;=\;1}^{T_{i}}x_{ti}\text{ and }{\bar{y}}_{\cdot i}=\frac{1}{T_{i}}\sum_{i\;=\;1}^{T_{i}}y_{ti}.$$

Then the between-person or *between-cluster* slope β_*B*_ can be obtained by analyzing these individual means using the regression equation

(12) $${\bar{y}}_{\cdot i}=\beta_{0}+\beta_{B}{\bar{x}}_{\cdot i}+e_{i}.$$

Additionally, the averaged within-person or *within-cluster* slope β_*W*_ can be obtained through applying CMC to both the predictor and the outcome variable, and analyze these data for individuals simultaneously, that is

(13) $$y_{ti}-{\bar{y}}_{\cdot i}=\beta_{W}(x_{ti}-{\bar{x}}_{\cdot i})+e_{ti}.$$

Clearly, β_*W*_ and β_*B*_ need not be the same.

Raudenbush and Bryk (2002) discuss how the slopes from diverse multilevel approaches are related to these two basic slopes. There are three approaches that can be used, that is: no centering (NC), grand-mean centering (GMC), and cluster-mean centering. GMC is simply a linear transformation of the data, and leads to a model that is statistically equivalent to NC (cf. Kreft et al., 1995; Raudenbush and Bryk, 2002; Snijders and Bosker, 2012). Therefore, we do not discuss this approach separately, and only focus on the comparison between NC and CMC below.

The model based on NC—with a random intercept and a fixed slope—can be expressed as

(14) $$\begin{array}{l} y_{ti}=\alpha_{i}^{n}+\beta_{i}^{n}x_{ti}+e_{ti}\\ \alpha_{i}^{n}=\gamma_{00}^{n}+u_{0i}^{n}\\ \beta_{i}^{n}=\gamma_{10}^{n}. \end{array}$$

whereas the corresponding model based on CMC of the predictor results in

(15) $$\begin{array}{l} y_{ti}=\alpha_{i}^{c}+\beta_{i}^{c}(x_{ti}-{\bar{x}}_{\cdot i})+e_{ti}\\ \alpha_{i}^{c}=\gamma_{00}^{c}+u_{0i}^{c}\\ \beta_{i}^{c}=\gamma_{10}^{c}. \end{array}$$

Note that the fixed slope γ^*c*^_10_ from the CMC model is analogous to β_*W*_ in Equation 13, with *y*_*ti*_ − *y*_· *i*_ being replaced by *y*_*ti*_ − α^*c*^_*i*_. Hence, CMC leads to an estimate of the within-cluster slope. The question is whether the within-cluster slope can also be obtained from the model in Equation 14.

To this end, we enter the level 2 expressions into the level 1 expressions, such that the model based on NC can be expressed as

(16) $$y_{ti}\;=\gamma_{00}^{n}+\gamma_{10}^{n}x_{ti}+u_{0i}^{n}+e_{ti},$$

and the model based on CMC can be expressed as

(17) $$y_{ti}\;=\gamma_{00}^{c}+\gamma_{10}^{c}x_{ti}-\gamma_{10}^{c}{\bar{x}}_{\cdot i}+u_{0i}^{c}+e_{ti}.$$

From these expressions it becomes clear that these models are not equivalent, as one cannot be considered an alternative parametrization of the other (cf. Kreft et al., 1995). This also implies that the within-cluster slope cannot be derived based on the results obtained from NC. Raudenbush and Bryk (2002) indicate that the slope of the level 1 predictor obtained with NC (γ^*n*^_10_) is “an uninterpretable blend” (p. 139) of the averaged within-cluster slope β_*W*_ and the between-cluster slope β_*B*_. This has led them to formulate the advice to use CMC whenever the interest is in obtaining an unbiased estimate of the within-cluster relationship, and the within-cluster and between-cluster relationships are expected to differ from each other (see also Enders and Tofighi, 2007).^3^

One could argue that the models above are not correct, because the between-cluster relationship is not explicitly modeled. Raudenbush and Bryk (2002) discuss the option of obtaining estimates of both β_*W*_ and β_*B*_ in a single multilevel model, through including the cluster means on the predictor as a level 2 predictor for the intercept. In case of NC, this results in

(18) $$\begin{array}{l} y_{ti}=\alpha_{i}^{n}+\beta_{i}^{n}x_{ti}+e_{ti}\\ \alpha_{i}^{n}=\gamma_{00}^{n}+\gamma_{01}^{n}{\bar{x}}_{\cdot i}+u_{0i}^{n},\\ \beta_{i}^{n}=\gamma_{10}^{n}. \end{array}$$

while in case of CMC this gives

(19) $$\begin{array}{l} y_{ti}=\alpha_{i}^{c}+\beta_{i}^{c}(x_{ti}-{\bar{x}}_{\cdot i})+e_{ti}\\ \alpha_{i}^{c}=\gamma_{00}^{c}+\gamma_{01}^{c}{\bar{x}}_{\cdot i}+u_{0i}^{c},\\ \beta_{i}^{c}=\gamma_{10}^{c}. \end{array}$$

In the latter approach, the within-cluster slope is again represented by γ^*c*^_10_, and now the between-cluster slope is represented by γ^*c*^_01_.

To see whether NC and CMC lead to equivalent models in this case, we substitute the level 2 expressions in the level 1 expression. For NC this results in

(20) $$y_{ti}\;=\gamma_{00}^{n}+\gamma_{01}^{n}{\bar{x}}_{\cdot i}+\gamma_{10}^{n}x_{ti}+u_{0i}^{n}+e_{ti},$$

and for CMC it results in

(21) $$y_{ti}\;=\gamma_{00}^{c}+(\gamma_{01}^{c}-\gamma_{10}^{c}){\bar{x}}_{\cdot i}+\gamma_{10}^{c}x_{ti}+u_{0i}^{c}+e_{ti},$$

showing the models are equivalent. Furthermore, it becomes clear that actually both models provide an estimate of the within-cluster slope, that is, γ^*n*^_10_ = γ^*c*^_10_ = β_*W*_. Additionally, we have γ^*n*^_01_ = γ^*c*^_01_ − γ^*c*^_10_ = β_*B*_ − β_*W*_, that is, γ^*n*^_01_ represents the *difference* in the between-cluster and the within-cluster slopes. This is also referred to as the *contextual* or *compositional effect* (cf. Raudenbush and Bryk, 2002 p. 141).^4^, and these models are referred to as contextual models.

In sum, when there is a fixed slope, NC, and CMC lead to equivalent models if one includes the cluster means for the level 1 predictor as a level 2 predictor for the intercept (Kreft et al., 1995). However, if the cluster means are not included, these two models are not equivalent.

### 2.2. Effects of NC and CMC when there is a random slope

While the model equivalence above is interesting, it is of limited value in practice, as we are often interested in models with random slopes. For instance, consider the middle panel of Figure 1, representing the hypothetical relationship between the number of negative events and negative affect in daily measurements: It shows that the strength of the within-person relationship differs across individuals.

Snijders and Bosker (2012) show that if there is a random slope, the model equivalence presented above no longer holds. Allowing for a random slope in the NC model in Equation 18, implies we have β^*n*^_*i*_ = γ^*n*^_10_ + *u*^*n*^_1*i*_, and we can thus write

(22) $$y_{ti}\;=\gamma_{00}^{n}+\gamma_{01}^{n}{\bar{x}}_{\cdot i}+\gamma_{10}^{n}x_{ti}+u_{1i}^{n}x_{ti}+u_{0i}^{n}+e_{ti}.$$

For the CMC model in Equation 19, a random slope implies we have β^*c*^_*i*_ = γ^*c*^_10_ + *u*^*c*^_1*i*_, such that the model can be expressed as

(23) $$\begin{array}{l} y_{ti}=\gamma_{00}^{c}+(\gamma_{01}^{c}-\gamma_{10}^{c}){\bar{x}}_{\cdot i}+\gamma_{10}^{c}x_{ti}+u_{1i}^{c}x_{ti}\\ \;-\;u_{1i}^{c}{\bar{x}}_{\cdot i}+u_{0i}^{c}+e_{ti}. \end{array}$$

This shows that—once there is a random slope—these models are no longer statistically equivalent, as they differ with respect to the term (−*u*^*c*^_1*i*_*x*_· *i*_). However, Kreft et al. (1995) pointed out that the fixed effect within-cluster slope is still the same across these two models: That is, γ^*n*^_10_ = γ^*c*^_10_ = β_*W*_ (see Kreft et al., 1995, p. 13). Hence, when the goal is to obtain an estimate of the within-cluster slope, and the within-cluster and between-cluster slope are expected to differ, it seems that one can chose either use CMC, or the contextual versions of CMC or NC/GMC: Although the contextual models are not equivalent when a random slope is included, they will result in the same within-cluster slope estimate.

For the sake of completeness, we also provide the expression for the models that include a random slope but without the cluster means as a level 2 predictor—as these are more common than the contextual models and the fixed slope models discussed above. In that case, NC leads to

(24) $$y_{ti}\;=\gamma_{00}^{n}+\gamma_{10}^{n}x_{ti}+u_{1i}^{n}x_{ti}+u_{0i}^{n}+e_{ti}.$$

and CMC leads to

(25) $$y_{ti}\;=\gamma_{00}^{c}+\gamma_{10}^{c}x_{ti}-\gamma_{10}^{c}{\bar{x}}_{\cdot i}+u_{1i}^{c}x_{ti}-u_{1i}^{c}{\bar{x}}_{\cdot i}+u_{0i}^{c}+e_{ti}.$$

As expected based on what was discussed above, both the fixed and the random parts of these models differ, and only CMC leads to an estimate of the average within-cluster slope, while NC leads to a slope that represents some mix of the within-cluster and between-cluster slopes.

### 2.3. Preliminary thoughts on centering in the multilevel autoregressive model

Before turning to our simulation study, we speculate briefly on the effects of NC and CMC in case of the multilevel autoregressive model. The contextual model would imply that we include the within-person means as a predictor for the intercept at level 2, that is

(26) $$\begin{array}{l} y_{ti}=\mu_{i}+\phi_{i}(y_{t-1,i}-\mu_{i})+e_{ti}\\ \mu_{i}=\gamma_{00}+\gamma_{01}\mu_{i}+u_{0i}\\ \phi_{i}=\phi +u_{1i}, \end{array}$$

where ϕ = β_*W*_ is the average within-cluster relationship, and γ_01_ = β_*B*_ is the between-cluster relationship. Note however that now μ_*i*_ appears on both sides of the equality sign, and it follows that γ_01_ = 1, γ_00_ = 0 and *u*_0*i*_ = 0 (and subsequently σ^2^_*u*0_ = 0)^5^.

We can draw two conclusions from this. First, including the within-person means as a level 2 predictor in a multilevel autoregressive model is not logical, and therefore the results for contextual models presented above are less relevant in the current context. Second, the within-cluster slope will—without exception—differ from the between-cluster slope in multilevel autoregressive models: That is, while the between-cluster slope is (essentially) 1, the within-cluster slope is identical to the auto-correlation and will thus have to lie between -1 and 1 for a stationary process (Hamilton, 1994). This is illustrated in the right panel of Figure 1, which shows that the between-cluster slope is equal to 1, while the within-cluster slope (averaged across individuals) is smaller (in this case between 0 and 1).

Applying the reasoning offered by Raudenbush and Bryk (2002) and Enders and Tofighi (2007) about the effects of NC/GMC vs. CMC in case of standard multilevel models to the multilevel autoregressive model, one may thus be inclined to think that: (a) NC/GMC will result in an *overestimation* of the fixed effect (i.e., average) autoregressive parameter, since β_*W*_ < β_*B*_ = 1; and (b) CMC will remove the “contamination” of β_*B*_, such that the fixed effect autoregressive parameter adequately represents β_*W*_, that is, the (averaged or pooled) within-person autoregression ϕ. This would imply that CMC should be the preferred form of centering in a multilevel autoregressive model.

Note that we already discussed two other reasons for preferring CMC in case of the multilevel autoregressive model, that is, it allows us to model μ_*i*_ as a random effect, rather than the less meaningful *c*_*i*_ = μ_*i*_(1 − ϕ_*i*_), and it allows us to include predictors for μ_*i*_ (rather than for *c*_*i*_). Taken together, these seem very convincing reasons for preferring CMC over GMC/NC in a multilevel autoregressive model.

## 3. Simulations

We performed a series of simulations to investigate the effect of NC vs. CMC on the estimation of the within-cluster slope. We begin with the standard multilevel model to verify the claims made by Raudenbush and Bryk (2002) and Enders and Tofighi (2007), and to determine whether these also generalize to models with a random slope (as presented in Equations 24 and 25). Following this, we consider the effects of NC and CMC in the multilevel autoregressive model, both with a fixed and a random autoregressive parameter. In addition, we consider the effects of diverse factors, that is: sample sizes, the sign and strength of the autoregressive parameter, and a level 2 predictor for the autoregressive parameter. All our simulations were performed in R (R Development Core Team, 2009). To estimate the multilevel models, we used the function lmer() from the R-package lme4 (Bates and Sarkar, 2007).

### 3.1. Simulations for the standard multilevel model

We begin with simulating data from the standard multilevel model with different within-cluster and between-cluster slopes, using Equation 19 for a model with a fixed within-cluster slope, and Equation 23 for a model with a random within-cluster slope. Our specific interest is in obtaining an appropriate estimate of the within-cluster slope, when this differs from the between-cluster slope. Hence, we want to verify that when the cluster means are not included as a level 2 predictor, the slope estimate obtained with NC is indeed a blend of the within-cluster and between-cluster slopes, while CMC (based on Equation 15) leads to a pure within-cluster slope estimate.

We used the following model parameter values: (a) the variance of the predictor *x*_*ti*_ within each cluster is 1, and the variance of the cluster means between the clusters is also 1; (b) the fixed effect within-cluster slope γ_10_ is 0.3; (c) the standard deviation of the within-cluster slope β_*i*_ is either 0 (i.e., fixed slope only model), or 0.1 (i.e., random slope model); (d) the between-cluster slope γ_01_ is 1; (e) the grand mean γ_00_ is zero; (f) the level 1 residual variance σ^2^_*e*_ was either 1 or 3; and (g) the level 2 residual variance for the intercept σ^2^_*u*0_ was either 1 or 0. The reason we considered 0 as well here, is because this would make the model more comparable to the multilevel autoregressive model we consider later on (see Equation 26). We set the number of clusters to 100, and the number of observations per cluster to 20.

The results are presented in Table 2: It includes the OLS estimate of the between-cluster slope (based on Equation 12), the OLS estimate of the within-cluster slope (based on Equation 13), and the fixed effects slope obtained with CMC and with NC. These confirm the point made by Raudenbush and Bryk (2002) and Enders and Tofighi (2007): While CMC leads to a slope estimate that is almost identical to the OLS within-cluster estimate and which adequately represents the actual within-cluster slope, the estimate obtained with NC is a blend of the within-cluster and the between-cluster slopes. Specifically, if the level 2 residual variance (i.e., σ^2^_*u*0_) becomes smaller relative to the level 1 residual variance (i.e., σ^2^_*e*_), the slope estimate is more strongly affected by the between-cluster slope. Furthermore, the results are very similar for models and data without a random slope (left part of Table 2), and with a random slope (right part of Table 2).

**Table 2:** **Estimates for fixed effect slope γ_10_**.

**Estimation method**	**σ^**2**^_***u0***_**	**σ^**2**^_***e***_**	**Fixed slope only**	**Random slope**
OLS between	1	1	0.963	0.965
	0	1	0.966	0.967
	0	3	0.965	0.966
OLS within	1	1	0.300	0.299
	0	1	0.298	0.300
	0	3	0.299	0.302
CMC (sample)	1	1	0.300	0.299
	0	1	0.298	0.300
	0	3	0.299	0.303
NC	1	1	0.323	0.323
	0	1	0.372	0.373
	0	3	0.492	0.489

Mean point estimates for fixed effects slope γ_*10*_ in a standard multilevel model with either a fixed slope only (left; β_*i*_ = γ_*10*_), or with a random slope (right; β_*i*_ = γ_*10*_ + *u*_*1i*_). True fixed effect within-cluster slope is γ_*10*_ = *0.3*, and true between-cluster slope is γ_*01*_ = *1*. Number of observations per cluster is 20; number of clusters is 100; number of replications is 1000. Estimation methods are: OLS between and within (Equations 12 and 13); centering per cluster (CMC) using the sample mean; and no centering (NC).

### 3.2. Simulations for the multilevel autoregressive model

To determine whether the results reported above generalize to the multilevel autoregressive model, we considered the following scenarios. We simulated data using the model defined in Equations 1–4, with: (a) a fixed effects within-cluster slope of ϕ = 0.3; (b) a standard deviation of the individual within-cluster slope ϕ_*i*_ of either 0 (for a model with a fixed autoregressive parameter only) or 0.1 (for a model with a random autoregressive parameter); (c) a level 2 variance of the intercept μ_*i*_ of 1, 3 or 9; and (d) a grand mean of 0. We used the same number of observations as in the previous simulations, that is, 100 clusters (i.e., persons here) and 20 observations per cluster (i.e., repeated measurements here). The results based on 1000 replications are presented in Table 3.

**Table 3:** **Estimates for fixed effect autoregressive parameter ϕ**.

**Estimation method**	**σ^**2**^_***e***_**	**Fixed slope only**	**Random slope**
OLS within	1	0.230	0.233
	3	0.229	0.233
	9	0.228	0.233
CMC (sample)	1	0.231	0.229
	3	0.230	0.229
	9	0.229	0.229
NC	1	0.304	0.307
	3	0.304	0.306
	9	0.303	0.304

Mean point estimates for the fixed effects autoregressive parameter ϕ in a multilevel autoregressive model, with a fixed slope only (left; ϕ_*i*_ = ϕ), and with a random slope (right; ϕ_*i*_ = ϕ + *u*_*1i*_). True fixed effect autoregressive parameter (i.e., the true within-cluster slope) is ϕ = *0.3*. Number of observations per person is 20; number of persons is 100; number of replications is 1000. Estimation methods are: OLS within (Equation 13); centering per cluster (CMC) using the sample mean; and no centering (NC).

As before, CMC leads to estimates that are very close to the OLS within-cluster estimates. However, for the multilevel autoregressive model, these are biased: That is, they underestimate the actual fixed effect autoregressive parameter (i.e., estimated bias between 0.069 and 0.071 for CMC). Surprisingly, NC leads to an estimate that is less biased (i.e., estimated bias between 0.003 and 0.007). In Appendix 2, this downward bias for the OLS within-cluster estimate in multilevel autoregressive model is confirmed analytically. Note further that whether or not inertia was random, did not affect the results substantially.

### 3.3. Investigating the influence of other factors

To gain more insight in this matter, we considered three additional factors that may affect the estimation of the within-cluster slope in a multilevel autoregressive model. First, in addition to using the individual sample means in CMC (i.e., *y*_· *i*_), we also considered the empirical Bayes estimator (also referred to as shrinkage estimator) of the individuals' means (i.e., $\hat{\mu }$_*i*_, obtained with estimating the empty model first), and the true person means that were used to generate the data (i.e., μ_*i*_; we considered this option here to see to what extent the results for CMC can be attributed to having to use an estimate of the individual's mean). Second, we considered different samples sizes, both with respect to number of persons *N*, and the number of repeated measures *T*. Third, we considered different strengths and signs of the fixed effects autoregressive parameter. Throughout we used the level 1 residual variance σ^2^_*e*_ = 3, the level 2 intercept variance σ^2^_*u*0_ = 3, and the level 2 slope variance σ^2^_*u*1_ = 0.01.

Based on the results presented in Table 4, we can conclude the following. First, CMC of the autoregressive predictor leads to bias, regardless of the kind of mean that is used (i.e., the sample estimate *y*_· *i*_, the empirical Bayes estimate $\hat{\mu }$_*i*_, or the true value μ_*i*_). It is noteworthy that even using the true mean results in bias that is about the same as the bias obtained with the empirical Bayes estimate of the mean, while using the sample mean leads to only slightly more bias. In contrast, NC does not lead to (considerable) bias. Second, when using CMC, increasing the number of observations per person (i.e., *T*) leads to a decrease in bias, whereas the number of individuals *N* does not affect the bias. Third, the bias for CMC reported in Table 4 is *always negative*, regardless of the actual value of ϕ, although the bias is largest when ϕ = 0.3, and smallest when ϕ = −0.3. This implies that in general, ϕ will be underestimated when CMC is used, and the bias is larger when ϕ is positive (which will often be the case in practice). This is also confirmed by the analytical results in Appendix 2.

**Table 4:** **Bias and coverage rates for fixed autoregressive parameter ϕ in multilevel autoregressive model under diverse scenarios**.

**AR parameter**	**Sample size**		**Bias**				**CR_**0.95**_**			
	**N**	**T**	**NC**	**C(***y***_·*i*_)**	**C($\hat{\mu }$_***i***_)**	**C(μ_***i***_)**	**NC**	**C(***y***_·*i*_)**	**C($\hat{\mu }$_***i***_)**	**C(μ_***i***_)**
ϕ_*i*_ ~ *N*(0.3, 0.1)	20	20	0.002	−0.072	−0.069	−0.068	0.928	0.762	0.785	0.787
		50	0.000	−0.027	−0.027	−0.026	0.940	0.900	0.901	0.898
		100	0.000	−0.013	−0.013	−0.013	0.932	0.932	0.932	0.932
	50	20	0.005	−0.071	−0.069	−0.067	0.893	0.480	0.512	0.518
		50	0.001	−0.027	−0.026	−0.026	0.936	0.800	0.804	0.805
		100	0.000	−0.013	−0.013	−0.013	0.946	0.902	0.902	0.903
	100	20	0.006	−0.070	−0.068	−0.066	0.892	0.196	0.227	0.242
		50	0.001	−0.027	−0.027	−0.027	0.930	0.623	0.630	0.637
		100	0.000	−0.013	−0.013	−0.013	0.930	0.851	0.854	0.851
ϕ_*i*_ ~ *N*(0, 0.1)	20	20	0.001	−0.053	−0.050	−0.050	0.923	0.844	0.858	0.851
		50	−0.000	−0.020	−0.020	−0.020	0.944	0.912	0.915	0.911
		100	0.000	−0.010	−0.009	−0.009	0.929	0.926	0.926	0.925
	50	20	0.003	−0.052	−0.049	−0.049	0.922	0.700	0.727	0.725
		50	−0.001	−0.021	−0.021	−0.021	0.942	0.860	0.862	0.861
		100	0.000	−0.010	−0.010	−0.010	0.939	0.910	0.910	0.909
	100	20	0.003	−0.053	−0.051	−0.050	0.929	0.431	0.479	0.477
		50	0.000	−0.021	−0.021	−0.021	0.931	0.775	0.785	0.785
		100	0.000	−0.010	−0.010	−0.010	0.942	0.892	0.896	0.896
ϕ_*i*_ ~ *N*(−0.3, 0.1)	20	20	0.003	−0.034	−0.031	−0.032	0.943	0.907	0.916	0.913
		50	0.000	−0.014	−0.013	−0.014	0.940	0.932	0.934	0.928
		100	0.001	−0.005	−0.005	−0.005	0.928	0.928	0.929	0.929
	50	20	0.000	−0.038	−0.035	−0.036	0.940	0.783	0.802	0.795
		50	0.000	−0.014	−0.014	−0.014	0.932	0.894	0.896	0.896
		100	0.000	−0.007	−0.006	−0.006	0.927	0.914	0.914	0.914
	100	20	0.000	−0.039	−0.036	−0.037	0.932	0.597	0.639	0.624
		50	0.000	−0.015	−0.015	−0.015	0.928	0.848	0.851	0.851
		100	0.000	−0.007	−0.007	−0.007	0.942	0.908	0.911	0.911

Bias and coverage rates of 95% confidence intervals (CR_*0.95*_) based on 1000 replications. *N* refers to number of persons, *T* refers to number of observations per person. The random coefficient ϕ_*i*_ comes from a normal distribution, with mean ϕ (either 0.3, 0, or −0.3), and standard deviation 0.1. Results are obtained for: NC of the autoregressive predictor; C(y_· *i*_) is CMC using the sample mean; C($\hat{\mu }$_*i*_) is CMC using the empirical Bayes estimate; and C(μ_*i*_) is CMC using the true mean per person (for comparison).

With respect to the coverage rates of the 95% confidence intervals, we make the following two observations. First, while in general they are too low, for NC most coverage rates are above 0.900, while for all three forms of CMC they are much lower (which is not surprising, given the bias of CMC estimates). Second, while increasing *T* leads to higher coverage rates for the CMC approaches, increasing *N* actually leads to lower coverage rates. This result is explained by the fact that the standard errors decrease when *N* increases, while the bias remains unaffected by changes in *N*. Note that the pattern for the coverage rates obtained with NC is less clear.

### 3.4. Including a level 2 predictor of the autoregressive parameter

An important question when applying the multilevel autoregressive model is whether other variables predict individual differences in the autoregression (cf. Suls et al., 1998; Kuppens et al., 2010). Therefore, we performed an additional simulation study to determine the effect of CMC and NC on the estimation of the effect of a level 2 predictor on the autoregressive parameter.

Let *z*_*i*_ be a level 2 predictor that may have an effect on the individuals' average score μ_*i*_, but more importantly, may have an effect on the individuals' autoregressive parameter ϕ_*i*_. We assume this level 2 predictor is centered across people. When using NC, the model can be expressed as

(27) $$\begin{array}{l} y_{ti}=c_{i}+\phi_{i}^{n}y_{t-1,i}+e_{ti}\\ c_{i}=\gamma_{00}^{n}+\gamma_{01}^{n}z_{i}+u_{0i}\\ \phi_{i}^{n}=\gamma_{10}^{n}+\gamma_{11}^{n}z_{i}+u_{1i} \end{array}$$

where γ^*n*^_00_ is the overall intercept, and γ^*n*^_10_ is the average autoregressive parameter (assuming the level 2 predictor *z*_*i*_ is centered). The regression coefficients γ^*n*^_01_ and γ^*n*^_11_ represent the effects of the level 2 predictor on the individuals' intercept *c*_*i*_ and their autoregressive parameter ϕ^*n*^_*i*_, respectively.

In contrast, when using CMC for the autoregressive predictor, the model can be defined as

(28) $$\begin{array}{l} y_{ti}=\mu_{i}+\phi_{i}^{c}(y_{t-1,i}-\mu_{i})+e_{ti}\\ \mu_{i}=\gamma_{00}^{c}+\gamma_{01}^{c}z_{i}+u_{0i}\\ \phi_{i}^{c}=\gamma_{10}^{c}+\gamma_{11}^{c}z_{i}+u_{1i} \end{array}$$

where γ^*c*^_00_ now represents the grand mean, and γ^*c*^_10_ is again the average autoregressive parameter (assuming the level 2 predictor *z*_*i*_ is centered). The regression coefficients γ^*c*^_01_ and γ^*c*^_11_ represent the effects of the level 2 predictor on the individuals' means μ_*i*_ and their autoregressive parameters ϕ^*c*^_*i*_, respectively.

Based on the results from the previous simulations, we expect that CMC (as in Equation 28) will lead to a downward bias in the estimation of the average autoregressive parameter ϕ (i.e., γ^*c*^_10_ will be an underestimate), while NC (as in Equation 27) is not associated with such bias (i.e., γ^*n*^_10_ is an unbiased estimate of ϕ). However, the question here is how CMC and NC affect the estimation of the level 2 predictor on ϕ_*i*_, that is, γ^*c*^_11_ and γ^*n*^_11_.

We created a level 2 predictor with a mean of zero and a variance of 0.01. We chose this rather small variance for numerical reasons: Because the variance of ϕ_*i*_ is necessarily small (say about 0.01), having a level 2 predictor with a large variance may lead to numerical problems in estimating the regression coefficient γ_11_. The mean autoregressive parameter ϕ was set to 0.3. The effect of the level 2 predictor *z*_*i*_ on the individual inertia parameters ϕ_*i*_ was set to 0.4. The other parameters were chosen such that the correlations between μ_*i*_, ϕ_*i*_ and *z*_*i*_ were not unrealistically high (μ_*i*_ and ϕ_*i*_ were both correlated 0.37 with *z*_*i*_, and 0.14 with each other). After generating μ_*i*_ and ϕ_*i*_ from *z*_*i*_, we used Equations 1 and 3 to generate the data. The results for this simulation study are presented in Table 5.

**Table 5:** **Results for average autoregressive parameter ϕ and the effect of a level 2 predictor *z*_*i*_ on the autoregressive parameter ϕ_*i*_**.

**N**	**T**	**Bias**				**CR_**0.95**_**			
		**γ_10_**		**γ_11_**		**γ_10_**		**γ_11_**	
		**NC**	**CMC**	**NC**	**CMC**	**NC**	**CMC**	**NC**	**CMC**
20	20	−0.007	−0.076	−0.050	−0.007	0.897	0.720	0.944	0.958
	50	−0.005	−0.030	−0.045	−0.026	0.922	0.856	0.939	0.944
	100	0.000	−0.013	−0.019	−0.008	0.923	0.909	0.942	0.944
50	20	0.004	−0.071	−0.068	−0.022	0.885	0.476	0.950	0.959
	50	0.001	−0.026	−0.032	−0.014	0.904	0.781	0.945	0.948
	100	0.000	−0.013	−0.018	−0.006	0.924	0.890	0.953	0.949
100	20	0.004	−0.071	−0.084	−0.036	0.918	0.170	0.921	0.940
	50	0.000	−0.027	−0.024	−0.003	0.907	0.628	0.944	0.955
	100	0.001	−0.012	−0.019	−0.008	0.928	0.832	0.939	0.942

Bias and coverage rate of 95% confidence intervals (CR_*0.95*_) based on 1000 replications. Results for γ_*10*_ = *0.3*, that is, the average autoregressive parameter ϕ, and for γ_*11*_ = *0.4*, that is, the effect of a level 2 predictor on the autoregressive parameter, using NC and CMC (with sample mean) for the autoregressive predictor.

The left part of the Table 5 contains the results that reflect the bias. In line with our previous results, the average autoregressive parameter γ_10_ = ϕ is characterized by a downward bias when CMC is used for the autoregressive predictor, while NC leads to unbiased estimates. However, when considering the effect of CMC vs. NC on the estimation of γ_11_, we see that CMC actually leads to *less bias* than NC. Note also that while increasing *T* reduces the bias obtained with NC, the effect of increasing *N* is not that clear (i.e., when *T* = 20, increasing *N* actually increases the bias).

The right part of Table 5 contains the coverage rates of the 95% confidence intervals. As before, the coverage rates for the average autoregressive parameter obtained with CMC are lower than those obtained with NC. For γ_11_ the coverage rates obtained with NC are in general lower than those obtained with CMC (which was to be expected given the results for the bias).

### 3.5. Conclusion

The first set of simulations presented in this section clearly illustrated the point made by Raudenbush and Bryk (2002) and Enders and Tofighi (2007) in case of a standard multilevel model. In addition it was shown that the claims regarding the within-cluster slope generalize to the model with a random slope, in that CMC leads to an estimate of the within-cluster slope, whereas NC results in a blend of the within-cluster and the between-cluster slope. The second set of simulations was based on the multilevel autoregressive model and showed that while CMC still leads to results that are almost identical to the OLS-within estimate, both of these are biased with respect to the actual within-cluster slope (i.e., the autoregressive relationship).

Additional simulations showed that there is a downward bias regardless of the sign of ϕ, and that this bias is most severe when *T* is small, while *N* has little (if any) influence. This bias could not be attributed to the quality of the estimate of the individuals' means (as very similar results are obtained when using the true means μ_*i*_ for centering). Furthermore, these results were supported by the derived relationship between the OLS within-cluster slope estimate and the value of ϕ in Appendix 2. In contrast, NC does not lead to bias in the estimation of the autoregressive parameter, which implies that the obtained result is actually *not contaminated* by the between-cluster relationship, as is the case in regular multilevel analysis. Finally, when adding a level 2 predictor to the model, the results described above for the average autoregressive parameter remain intact, but for the effect of the level 2 predictor on the autoregressive parameter, NC actually results in bias, whereas CMC does not.

## 4. Application: part 2

Returning to the empirical data that we introduced in the beginning of this paper, we are now able to study inertia in daily relationship specific PA and NA, and include a level 2 predictor for the individual differences in the means and the inertia. We used Relationship Satisfaction, which was obtained prior to the diary study, and standardized this level 2 predictor to facilitate interpretation (i.e., we subtracted the grand mean, and divided it by the grand standard deviation). We used the model based on CMC (see Equation 28), and summarized the results for all the fixed effects in Table 6. We also included the estimate of the fixed effects inertia obtained with NC in this table, as the simulations reported in this paper showed that this is an unbiased estimate of the average inertia, whereas the corresponding estimate obtained with CMC is negatively biased.

**Table 6:** **Results for multilevel autoregressive model with a level 2 predictor (with random effects)**.

		**Males**			**Females**		
		**est**.	**SD**	***t*-value**	**est**.	**SD**	***t*-value**
PA	γ^*c*^_00_	3.514	0.043	81.98	3.498	0.043	80.93
	γ^*c*^_01_	0.303	0.046	6.58	0.383	0.045	8.43
	γ^*c*^_10_	0.354	0.016	22.46	0.340	0.015	22.50
	γ^*n*^_10_	0.385	0.015	25.41	0.369	0.015	24.69
	γ^*c*^_11_	−0.045	0.017	−2.67	−0.015	0.016	−0.98
NA	γ^*c*^_00_	1.346	0.022	59.96	1.346	0.020	5.75
	γ^*c*^_01_	−0.072	0.024	−2.99	−0.132	0.022	−6.15
	γ^*c*^_10_	0.242	0.017	14.31	0.224	0.016	13.64
	γ^*n*^_10_	0.267	0.017	16.14	0.254	0.017	15.39
	γ^*c*^_11_	−0.046	0.017	−2.63	−0.060	0.016	−3.71

Parameter estimates, standard errors and t-values for the fixed effects parameters in a multilevel autoregressive model (with random intercept and slope), for males and females. Parameters include: (a) the grand mean (i.e., γ^*c*^_*00*_); (b) the effect of Relationship Satisfaction on the individuals' means (i.e., γ^*c*^_*01*_); (c) the average inertia obtained with CMC (i.e., γ^*c*^_*10*_), and with NC (i.e., γ^*n*^_*10*_); and (d) the effect of Relationship Satisfaction on the individuals' inertias (i.e., γ^*c*^_*11*_).

It shows that on average there is significant inertia in relationship specific PA and NA for both males and females (see γ^*n*^_10_). In addition, Relationship Satisfaction proved a significant positive predictor of mean levels of relationship specific PA in both males and females, and a significant negative predictor of mean levels of relationship specific NA in both males and females (see γ^*c*^_01_). Furthermore, Relationship Satisfaction is a significant negative predictor of inertia in relationship specific PA in males (but not in females), and in relationship specific NA in males and females (see γ^*c*^_11_). This implies that individuals who are less satisfied with their relationship, are characterized by more carryover of relationship specific NA, than individuals who are more satisfied with their relationship. In addition, males who are less satisfied with their relationship, are also characterized by more carryover in their relationship specific PA. While the latter may seem surprising at first—as it implies that elevated relationship specific PA tends to persist over time for males who are less satisfied with their relationship—it also implies that attenuated relationship specific PA tends to prevail, which could be considered undesirable. These results are in agreement with other findings regarding inertia reported by Koval et al. (2013), who found that the inertias of PA and NA are positively correlated, and Kuppens et al. (2012), who found that inertia of angry and dysphoric behavior, but also of happy behavior all predicted the onset of depression. Taken together, these results seem to confirm the idea that inertia—whether in pleasant or unpleasant emotions—is a detrimental property of affect regulation, reflective of some maladaptive process.

## 5. Discussion

Over the past two decades we have witnessed an exponential increase in the number of studies based on intensive longitudinal data in the social sciences. This development is triggered by the rapid development of electronic data collection methods based on hand-held computers, the internet, and—more recently—smart phones (Trull and Ebner-Priemer, 2013): As a result it has become relatively easy to gather large numbers of repeated measurements from a large sample of individuals. Such data differ from more traditional longitudinal data in two important ways: (1) intensive longitudinal data contain many more measurements per individual (i.e., often *T* > 20) than traditional longitudinal data (i.e., often *T* < 10); and (2) the measurements in intensive longitudinal data are typically spaced relatively close to each other in time (e.g., measurements are taken at a daily basis using a daily diary method, or even multiple times a day using experience method sampling), whereas traditional longitudinal data are characterized by much larger intervals between measurements (e.g., annual measurements are not uncommon). These differences reflect a different focus on part of the researchers: Whereas the purpose of many studies based on traditional longitudinal data is to discover broad underlying increasing or decreasing trends, the purpose of studies based on intensive longitudinal data is to gain more insight into the patterns of fluctuations in affect, behavior, and cognition in daily life (Bolger et al., 2003; Mehl and Conner, 2012).

One particular aspect of such patterns is referred to as inertia or autoregression, and represents the amount of carryover from one measurement occasion to the next (Suls et al., 1998). Diverse empirical studies have now shown that individual differences in inertia are meaningful with respect to the way people differ in their regulation of emotions and behavior. As the popular method for studying inertia is through a multilevel autoregressive model, an important research question in this area is whether the autoregressive predictor included at level 1 should be centered per person or not.

The current study shows through a series of simulations that CMC should be preferred if: (a) one wishes to obtain a meaningful intercept (i.e., an intercept that represents the individual's mean score over time, which can be interpreted as his/her trait score); and (b) the interest is in how the autoregressive parameter depends on a level 2 predictor. However, CMC should *not* be used when the interest is in whether or not there is an autoregressive relationship *on average* (i.e., across individuals).

In practice, researchers using a multilevel autoregressive model to study inertia are likely to be interested in various aspects of the model, including the individuals' means, the average autoregressive parameter, and the effect of a level 2 predictor on the individuals' means and autoregressive parameters. In that case, it may be wise to use both estimation procedures, as we did in the empirical application, and to use CMC for the estimation of the grand mean and the effect of the level 2 predictor on the individual means and autoregressive parameters, while NC results are used for determining whether there is an autoregressive effect on average. While this may be unconventional advice, it is based on the rather clear simulation results presented in this paper.

Given the recent interest in inertia, and its emerging recognition as a separate and valuable property of regulation that is related to but does not coincide with more traditionally studied process features such as the tendency to ruminate or the persistence of negative thoughts, we expect to see more work in this area. Hence, it is important to improve our ways to estimate average inertia, and individual differences therein. Specifically, the current study has shown that many of the inertia estimates reported in the literature may actually be underestimates of the true inertias, simply because the lagged autoregressive predictor was centered per person (e.g., Koval et al., 2012; Brose et al., 2014). Although this may not come as a surprise to those familiar with time series literature, as it has been known for a long time that estimates of autoregressive parameters are biased (cf., Orcutt, 1948; Marriott and Pope, 1954), it is an unexpected result from a multilevel perspective. Furthermore, it is of interest that the bias disappears when the lagged autoregressive predictor is not centered; in fact, this may be considered an important advantage of the multilevel approach over a two-step procedure in which during the first step individual time series models are estimated, while in the second step the individual parameters are combined into a population model.

Additional improvements in the study of inertia may come from taking measurement error into account—which is also likely to obscure the actual inertia of a process—and developing appropriate techniques for handling unequal intervals between the observations—which are a feature of certain intensive longitudinal data, and which may lead to less precise estimates when not taken into account, and therefor to more difficulty in detecting relationships between inertia and other person characteristics. When these issues are handled in an appropriate way, inertia may prove to be an even more important feature of regulatory processes in psychology than the existing studies already suggest.

Finally, note that the advice given here regarding CMC vs. NC or GMC *exclusively* applies to an autoregressive level 1 predictor: That is, if one includes other level 1 predictors, the common results based on Raudenbush and Bryk (2002) apply to them, meaning that CMC of these predictors should be preferred over NC or GMC if the within-cluster and between-cluster slopes are expected to differ, and one wants to obtain an estimate of the within-cluster slope.

### Conflict of interest statement

The authors declare that the research was conducted in the absence of any commercial or financial relationships that could be construed as a potential conflict of interest.

## Figures and Tables

**Figure A1:**
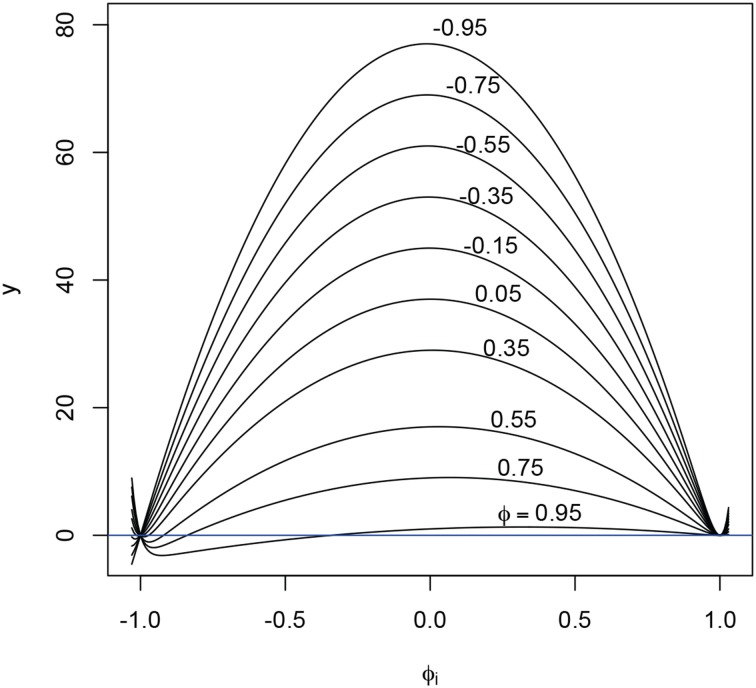
**Numerator of expectation, [that is, *y* = *T*(1 − ϕ)(1 − ϕ^2^_*i*_) − (1 − 2ϕϕ_*i*_ + ϕ^2^_*i*_)(1 − ϕ^*T*^_*i*_)] plotted against ϕ_*i*_, for *T* = 40 and different values of ϕ (i.e., average ϕ_*i*_)**. Note that only for ϕ = 0.95 the numerator becomes negative on a substantial portion of the interval (−1, 1). See text for implications.

## Footnotes

^1^As we will show later on, the between-person slope will always be (essentially) 1 in this model, while the within-person slope is expected to lie between −1 and 1.

^2^Note that the model presented in Equations 1 and 2 corresponds to what is known as a random intercept model or empty model, and is typically considered as one of the options in longitudinal multilevel modeling.

^3^Note that there may be situations in which the within-person slope and the between-person slope do not differ that much, such that failing to separate them does not affect the results very much: For instance, in the middle panel of Figure 1 the relationship between negative events and negative affect is represented, which shows that within individuals, there is (on average) a positive relationship (i.e., people tend to experience more negative affect on days that more negative events occur), and the between-person relationship is very similar (i.e., people who tend to experience more negative events on average, also tend to have higher levels of negative affect on average). In this case, using CMC or NC/GMC will not change the estimate of the fixed slope very much. However, the point remains that to obtain an adequate estimate of the averaged within-cluster slope, CMC should be preferred.

^4^Here it represents the expected difference in number of typos when comparing two participants who type the same number of words, while they differ one unit on the number of words they type per minute on average (meaning they have different levels of experience).

^5^In practice, the means on the outcome variable *y*_*ti*_ are virtually identical to the cluster means on the predictor *y*_*t* − 1, *i*_, as it is the same variable; slight difference may arise however, because the outcome runs over *t* = 2, …, *T*_*i*_ while the predictor runs over *t* = 1, …, *T*_*i*_ − 1. As *T* becomes larger, these differences will become smaller.

## Appendix 1

To show that the model expressed in Equations 5, 6 and 7, is structurally equivalent to the model in Equations 1–4, we first make use of the fact that *y*_*t* − 1, *i*_ − μ_*i*_ = *a*_*t* − 1, *i*_, such that we can rewrite Equation 3 as

(A1) $$\begin{array}{l} a_{ti}=\phi_{i}a_{t\;-\;1,i}+e_{ti}\\ \;=\phi_{i}(y_{t\;-\;1,i}-\mu_{i})+e_{ti}\\ \;=\phi_{i}y_{t\;-\;1,i}-\phi_{i}\mu_{i}+e_{ti}. \end{array}$$

Entering this in Equation 1, we can write

(A2) $$\begin{array}{l} y_{ti}=\mu_{i}-\phi_{i}\mu_{i}+\phi_{i}y_{t\;-\;1,i}+e_{ti}\\ \;=(1-\phi_{i})\mu_{i}+\phi_{i}y_{t\;-\;1,i}+e_{ti}, \end{array}$$

which shows that

(A3) $$c_{i}=(1-\phi_{i})\mu_{i}.$$

This is a standard result from the time series literature on the first-order autoregressive model (cf. Hamilton, 1994).

## Appendix 2

The rather unexpected results regarding CMC (i.e., centering the autoregressive predictor per person actually leads to bias in estimating the autoregressive parameter), was confirmed by the near identical results when using OLS. Below we show that OLS indeed leads to bias in the estimation of the autoregressive parameter ϕ. For simplicity of the presentation we will not make notational distinction between a random variable and it's (observed) value here.

Note that the OLS estimate of the regression model *y*_*i*_ = *b*_0_ + *b*_1_ *x*_*i*_ + *e*_*i*_ can be expressed as $\hat{b}$_1_ = cov(*x*_*i*_, *y*_*i*_)/var(*x*_*i*_). In a similar fashion, the OLS estimate of the within-person relationship ϕ in the autoregressive multilevel model can be expressed as the covariance between the person-centered predictor variable *y*_*ti*_ and the person-centered outcome variable *y*_*i*, *t* + 1_, divided by the variance of the person-centered predictor variable. To this end, let *T*^*^_*i*_ = *T*_*i*_ − 1, and let

(A4) $${\bar{y}}_{i\cdot (1)}=\frac{1}{T_{i}^{*}}\sum_{t\;=\;1}^{T_{i}^{*}}y_{ti}\text{ and }{\bar{y}}_{i\cdot (2)}=\frac{1}{T_{i}^{*}}\sum_{t\;=\;1}^{T_{i}^{*}}y_{i,t\;+\;1}$$

represent the estimated person means of the predictor variable *y*_*ti*_ and the outcome variable *y*_*i*, *t* + 1_, respectively. Then the OLS estimator of ϕ can be expressed as

(A5) $$\begin{array}{l} \hat{\phi }=\frac{\sum_{i\;=\;1}^{n}\{\frac{1}{T_{i}^{*}}\sum_{t\;=\;1}^{T_{i}^{*}}\left(y_{ti}-{\bar{y}}_{i\cdot (1)})(y_{i,t\;+\;1}-{\bar{y}}_{i\cdot (2)})\right\}}{\sum_{i\;=\;1}^{n}\{\frac{1}{T_{i}^{*}}\sum_{t\;=\;1}^{T_{i}}{\left(y_{ti}-{\bar{y}}_{i\cdot (1)}\right)}^{2}\}}\\ \;=\frac{\frac{1}{n}\sum_{i\;=\;1}^{n}\{\frac{1}{T_{i}^{*}}\sum_{t\;=\;1}^{T_{i}\;-\;1}(y_{ti}y_{it\;+\;1}-T_{i}^{*}{\bar{y}}_{i\cdot (1)}{\bar{y}}_{i\cdot (2)})\}}{\frac{1}{n}\sum_{i\;=\;1}^{n}\{\frac{1}{T_{i}^{*}}\sum_{t\;=\;1}^{T_{i}}\left(y_{ti}^{2}-T_{i}^{*}{\bar{y}}_{i\cdot (1)}^{2})\right\}}, \end{array}$$

To derive the asymptotic bias of this estimator, we begin by deriving the numerator of Equation A5. To this end, we first consider the conventional estimate of the covariance between *y*_*ti*_ and *y*_*i*, *t* + 1_ per person, that is,

$$s_{i}(t+1,t)=\frac{1}{T_{i}^{*}}\sum_{t\;=\;1}^{T_{i}^{*}}\{y_{ti}y_{it\;+\;1}-T_{i}^{*}{\bar{y}}_{i\cdot (1)}{\bar{y}}_{i\cdot (2)}\}.$$

Taking the expectation of this covariance, conditional on *i*, gives

(A6) $$\begin{array}{l} E[s_{i}(t+1,t)|\;i]=\frac{1}{T_{i}^{*}}\overset{T_{i}^{*}}{\underset{t\;=\;1}{\sum }}\{E[y_{ti}y_{i,t\;+\;1}|\;i]\\ \;-T_{i}^{*}E[{\bar{y}}_{i\cdot (1)}{\bar{y}}_{i\cdot (2)}|\;i]\}. \end{array}$$

Focussing on the first expectation on the right-hand side of Equation A6, we make use of the fact that *y*_*i*, *t* + 1_ = *c*_*i*_ + ϕ_*i*_ *y*_*ti*_ + *e*_*i*, *t* + 1_, such that we can write

(A7) $$\begin{array}{l} E[y_{ti}y_{i,t\;+\;1}|i]=E[y_{ti}\{c_{i}+\phi_{i}y_{ti}+e_{i,t\;+\;1}\}|i]\\ \;=E[y_{ti}c_{i}|i]+E[\phi_{i}y_{ti}^{2}|i]+E[y_{ti}e_{i,t\;+\;1}|i]\\ \;=(1-\phi_{i})\mu_{i}E[y_{ti}|i]+\phi_{i}E[y_{ti}^{2}|i]\\ \;=(1-\phi_{i})\mu_{i}^{2}+\phi_{i}(\sigma_{i}^{2}+\mu_{i}^{2})\\ \;=\mu_{i}^{2}+\phi_{i}\sigma_{i}^{2}, \end{array}$$

where *c*_*i*_ = μ_*i*_(1 − ϕ_*i*_), μ_*i*_ = *E*(*y*_*ti*_|*i*) and $\sigma_{i}^{2}=\text{Var}(y_{ti}|i)\propto \frac{1}{1-\phi_{i}^{2}}$.

The second expectation on the right-hand side of Equation A6 can be rewritten (using the geometric series), to obtain

(A8) $$\begin{array}{l} E[{\bar{y}}_{i\cdot (1)}{\bar{y}}_{i\cdot (2)}|\;i]=\frac{1}{T_{i}^{*}T_{i}^{*}}\sum_{t\;=\;1}^{T_{i}^{*}}\overset{T_{i}^{*}}{\underset{\tau \;=\;1}{\sum }}E[y_{ti}y_{i,\tau \;+\;1}|\;i]\\ \;=\frac{1}{T_{i}^{*}T_{i}^{*}}\sum_{t\;=\;1}^{T_{i}^{*}}\sum_{u\;=\;1}^{T_{i}^{*}}\{\phi_{i}^{\left|t\;-\;u\;-\;1\right|}\sigma_{i}^{2}+\mu_{i}^{2}\}\\ \;=\sigma_{i}^{2}\;\frac{T_{i}^{*}(1-\phi_{i}^{2})-(1+\phi_{i}^{2})(1-\phi_{i}^{T_{i}^{*}})}{T_{i}^{*2}{\left(1-\phi_{i}\right)}^{2}}\\ \;=+\mu_{i}^{2}. \end{array}$$

Inserting the expression in Equations A7 and A8 in Equation A6, the expected value for the covariance conditional on person *i* can be expressed as

(A9) $$E[s_{i}(t+1,t)|\;i]=\sigma_{i}^{2}\;\{\phi_{i}-\frac{T_{i}^{*}(1-\phi_{i}^{2})-(1+\phi_{i}^{2})(1-\phi_{i}^{T_{i}^{*}})}{T_{i}^{*2}{\left(1-\phi_{i}\right)}^{2}}\}.$$

In a similar way, the expected value of the variance conditional on person *i* can be obtained, resulting in

(A10) $$E[s_{i}(t,t)|\;i]=\sigma_{i}^{2}\;\{1-\frac{T_{i}(1-\phi_{i}^{2})-2\phi_{i}(1-\phi_{i}^{T_{i}})}{T_{i}^{2}{\left(1-\phi_{i}\right)}^{2}}\}.$$

By the law of large numbers, as the number of participants *n* → ∞, the numerator on the right-hand side of Equation A5 converges in probability to *E*[*s*_*i*_(*t* + 1, *t*)] = *E*{*E*[*s*_*i*_(*t* + 1, *t*)| *i*]}, while the denominator converges in probability to *E*[*s*_*i*_(*t*, *t*)] = *E*{*E*[*s*_*i*_(*t*, *t*)| *i*]}. Therefore,

(A11) $$\hat{\phi }\overset{p}{\to }\frac{E[s_{i}(t+1,t)]}{E[s_{i}(t,t)]}=\frac{E[\sigma_{i}^{2}\{\phi_{i}-\frac{T_{i}^{*}(1-\phi_{i}^{2})-(1+\phi_{i}^{2})(1-\phi_{i}^{T_{i}^{*}})}{T_{i}^{*2}{\left(1-\phi_{i}\right)}^{2}}\}]}{E[\sigma_{i}^{2}\{1-\frac{T_{i}(1-\phi_{i}^{2})-2\phi_{i}(1-\phi_{i}^{T_{i}})}{T_{i}^{2}{\left(1-\phi_{i}\right)}^{2}}\}]}.$$

To show that in general the asymptotic bias will be negative, for simplicity, we assume *T*_*i*_ is large enough to treat *T*^*^_*i*_ ≈ *T*_*i*_. Then we have to show that

$$\frac{E[\sigma_{i}^{2}\{\phi_{i}-\frac{T_{i}^{*}(1-\phi_{i}^{2})-(1+\phi_{i}^{2})(1-\phi_{i}^{T_{i}^{*}})}{T_{i}^{*2}{\left(1-\phi_{i}\right)}^{2}}\}]}{E[\sigma_{i}^{2}\{1-\frac{T_{i}(1-\phi_{i}^{2})-2\phi_{i}(1-\phi_{i}^{T_{i}})}{T_{i}^{2}{\left(1-\phi_{i}\right)}^{2}}\}]}\leq \phi .$$

As the denominator is always positive^6^ when *T*_*i*_ ≥ 2, this is equivalent to showing that

$$\begin{array}{l} E[\sigma_{i}^{2}(\phi_{i}-\phi )]\\ \;+E[\sigma_{i}^{2}\frac{T_{i}(1-\phi )(1-\phi_{i}^{2})-(1-2\phi \phi_{i}+\phi_{i}^{2})(1-\phi_{i}^{T_{i}})}{T_{i}^{2}{\left(1-\phi_{i}\right)}^{2}}]\geq 0. \end{array}$$

The first term on the left-hand side is the sum of the covariance between autoregressive parameter ϕ_*i*_ and the variance of the series σ^2^_*i*_. Note that σ^2^_*i*_ = σ^2^_*e*_/(1 − ϕ^2^_*i*_), which implies that σ^2^_*i*_ and ϕ_*i*_ are correlated. For symmetric distributions of ϕ_*i*_ around ϕ, using a Taylor expansion of $\frac{\phi_{i}-\phi }{1-\phi_{i}^{2}}$, we can show^7^ that the correlation is positive if ϕ > 0 and negative if ϕ < 0. Therefore, assuming a density *f*_Φ_(ϕ_*i*_) that is (approximately) symmetric about ϕ, the first term should be deemed positive if ϕ > 0, and negative, if ϕ < 0.

Regarding the second term, since the denominator in this term is always positive, the only way this expected value can be negative is if on a substantial portion of the support of the density *f*_Φ_(ϕ) of ϕ_*i*_ the numerator is negative. Since no closed form expression for this region can be found in terms of *T* and ϕ, below we plot the numerator for *T* = 40 and different values of ϕ in Figure A1. The interval for ϕ is limited to (−1, 1) by the requirement of stationarity. The picture is slightly different for uneven *T*, but since the term 1 − ϕ^*T*^ only really matters near the edges of the interval, it has little effect on the global shape. It is clear that only for extreme values of ϕ (ϕ > 0.9) a substantial portion of the numerator is negative, but then also only at the lower end of the interval. This means that for any reasonable *f*_Φ_(ϕ) (which incidentally must have $\int_{-1}^{1}\phi f_{\Phi }(\phi )d\phi =E(\phi_{i})>0.9$ and therefore cannot have a large probability mass in the area where the numerator is negative), the numerator will be positive and hence the second term will be positive. As *T*_*i*_ grows larger, the always positive term *T*_*i*_(1 − ϕ)(1 − ϕ^2^_*i*_) in the numerator becomes much larger than the term (1 − 2ϕϕ + ϕ^2^)(1 − ϕ^*T*^). Hence for values of *T*_*i*_ larger than 40, the negative region of the numerator in the expectation vanishes. As a result, the estimator $\hat{\phi }$ will in most cases underestimate the real value of ϕ.

From Equation A11 it can be seen that as the lengths *T*_*i*_ of the observed series increase without bound, $\hat{\phi }$ converges in probability to *E*{(σ^2^_*i*_ + μ^2^_*i*_)ϕ_*i*_}/*E*{(σ^2^_*i*_ + μ^2^_*i*_)}. The disconcerting consequence is that the OLS estimator may be biased, even if an infinity number of samples is obtained!

### The case of σ^2^_*u*1_ = 0

In the first set of simulations for the multilevel autoregressive model, σ^2^_*u*1_ = 0—that is, all individuals were characterized by the same autoregressive parameter (i.e., ϕ_*i*_ = ϕ with probability 1). In this case, setting *T*_*i*_ = *T* without loss of generality, the above inequality simplifies to

$$\begin{array}{l} E[\sigma_{i}^{2}\frac{T(1-\phi )(1-\phi^{2})-(1-\phi^{2})(1-\phi^{T})}{T^{2}{\left(1-\phi \right)}^{2}}]\;\;\;\;\;\;\;\;\;\;\;\\ \;=(1-\phi^{2})\frac{T(1-\phi )-(1-\phi^{T})}{T^{2}{\left(1-\phi \right)}^{2}}\;E[\mu_{i}^{2}]\geq 0, \end{array}$$

where the first expectation dropped out because *E*[σ^2^_*i*_(ϕ_*i*_ − ϕ)] = 0 (since ϕ_*i*_ − ϕ = 0 when ϕ_*i*_ = ϕ).

The above inequality is satisfied if *g* = *T*(1 − ϕ) − (1 − ϕ^*T*^) ≥ 0. This is true for all − 1 ≤ ϕ ≤ 1, as then *g*′ = − *T* + *T*ϕ^*T* − 1^ ≤ 0 for all *T* = 1, 2, …, and *g* achieves a minimum of 0 at ϕ = 1. Hence, in this case, the estimator of ϕ is always biased downwards.
